# MiR-30c regulates cisplatin-induced apoptosis of renal tubular epithelial cells by targeting *Bnip3L* and *Hspa5*

**DOI:** 10.1038/cddis.2017.377

**Published:** 2017-08-10

**Authors:** Bin Du, Xiao-meng Dai, Shuang Li, Guo-long Qi, Guang-xu Cao, Ying Zhong, Pei-di Yin, Xue-song Yang

**Affiliations:** 1 Department of Pathology, Medical School, Jinan University, Guangzhou 510632, China; 2 Division of Medical Informatics, Medical School, Jinan University, Guangzhou 510632, China; 3 Division of Histology and Embryology, Key Laboratory for Regenerative Medicine of the Ministry of Education, Medical School, Jinan University, Guangzhou 510632, China

## Abstract

As a common anticancer drug, cisplatin has been widely used for treating tumors in the clinic. However, its side effects, especially its nephrotoxicity, noticeably restrict the application of cisplatin. Therefore, it is imperative to investigate the mechanism of renal injury and explore the corresponding remedies. In this study, we showed the phenotypes of the renal tubules and epithelial cell death as well as elevated cleaved-caspase3- and TUNEL-positive cells in rats intraperitoneally injected with cisplatin. Similar cisplatin-induced cell apoptosis was found in HK-2 and NRK-52E cells exposed to cisplatin as well. In both models of cisplatin-induced apoptosis *in vivo* and *in vitro*, quantitative PCR data displayed reductions in miR-30a-e expression levels, indicating that miR-30 might be involved in regulating cisplatin-induced cell apoptosis. This was further confirmed when the effects of cisplatin-induced cell apoptosis were found to be closely correlated with alterations in miR-30c expression, which were manipulated by transfection of either the miR-30c mimic or miR-30c inhibitor in HK-2 and NRK-52E cells. Using bioinformatics tools, including TargetScan and a gene expression database (Gene Expression Omnibus), *Adrb1*, *Bnip3L*, *Hspa5* and *MAP3K12* were predicted to be putative target genes of miR-30c in cisplatin-induced apoptosis. Subsequently, *Bnip3L* and *Hspa5* were confirmed to be the target genes after determining the expression of these putative genes following manipulation of miR-30c expression levels in HK-2 cells. Taken together, our current experiments reveal that miR-30c is certainly involved in regulating the renal tubular cell apoptosis induced by cisplatin, which might supply a new strategy to minimize cisplatin-induced nephrotoxicity.

Acute kidney injury (AKI) is an abrupt kidney failure or kidney damage within a few hours or a few days. AKI results in an accumulation of metabolic waste products in the blood and an imbalance of body fluid. AKI can also lead to many complications in other organs such as the brain, heart and lungs, and eventually, it often causes multiple organ failure.^1^ On the basis of the derivation of the AKI, AKI can be divided into hospital-acquired AKI (HA-AKI) and community-acquired AKI.^2, 3^ In China, instances of HA-AKI have markedly increased in the past two decades.^2^ The mortality of patients who die from AKI is as high as 8–9% of the total mortality in some Chinese hospitals, and, furthermore, the risk of a patient’s death is correlated with the degree of AKI severity.^4, 5^ According to the localization of the AKI pathogenesis, we can also classify HA-AKI as prerenal, postrenal or intrarenal. The principal cause of AKI is acute tubular injury although, and occasionally, acute vascular, glomerular and interstitial factors become pathogenic. Most of the AKI prognoses are not optimistic. Some literature has reported that the 10-year survival rate after suffering from AKI was less than 50%.^6, 7^ Of course, the long-term outcome after AKI is dependent on comorbid factors, causes of the initial disease and the patient’s age.

Unfortunately, the epidemiology of AKI has still remained vague.^1^ However, there is no doubt that most of the cases of HA-AKI are derived from the usage of nephrotoxic drugs in the clinic. Cisplatin (cis-DDP, CDDP) is certainly one of those nephrotoxic drugs that is commonly used in the clinic. Cisplatin, or cisplatinum, is a chemotherapeutic drug recognized for its use in many cancer treatments, including reproductive, bladder, head–neck and lung cancers, but its undesirable side effects include severe kidney problems.^8^ This is partly because of the characteristics of kidney function. We know that the function of the kidney includes filtering blood and absorbing minerals to produce urine as well as producing hormones, through which the kidney has a very important role in maintaining hemostasis of water and sodium in the body. Cisplatin is very soluble in water, and it can damage DNA structure and interfere with DNA replication and transcription through its high DNA-binding ability.^9^ The accumulation of cisplatin in the kidney, especially in the proximal renal tubules, is much higher than that in other organs in the human body.^10^ Flattened and deciduous renal epithelium, as well as dilated and transparent renal tubular lumina, are the pathological features of AKI in renal tubular epithelial cells.^11^ The pathophysiology of the renal tubular damage caused by cisplatin is thought to be induced by the following processes: (1) damage of proximal renal tubules; (2) oxidation stress; (3) inflammation; and (4) renal vascular damage. The reasons for the damage of the proximal renal tubules are considered to be because of cell apoptosis,^12^ dysfunctional autophagy,^13^ abnormal regulation of cell cycle proteins,^14^ MAPK signaling activation,^14^ DNA damage^15^ and dysfunctional mitochondria.^16^ In addition to cell apoptosis, cell necrosis contributes to the cell death in renal tubules induced by cisplatin. Whether or not necrosis or apoptosis occurs depends on the concentration of cisplatin. A high concentration (>800 *μ*mol/l) of cisplatin causes necrosis, while a low concentration (<800 *μ*mol/l) leads to apoptosis of proximal tubule cells.^17^ Accumulating evidence indicates that cell apoptosis is the main pathological cause for cisplatin-induced renal tubular damage^18^ although the underlying molecular mechanisms are still elusive.

Because the side effects of cisplatin have been well noted, some replacements have been developed. However, as one of the most common and efficient broad chemotherapeutic drugs in the clinic, cisplatin certainly cannot be completely replaced at the moment simply because its replacements have not been as effective.^19^ Thus, it will be necessary to explore novel strategies to minimize cisplatin-induced nephrotoxicity. MicroRNAs are small non-coding RNA molecules (~22 nucleotides) that have key roles in the regulation of gene expression during cell proliferation, differentiation, apoptosis and metabolism.^20^ The ways that microRNAs act on cells mainly have been considered to be through (1) degrading mRNA via complete complementary binding, (2) inhibiting mRNA translation via incomplete complementary binding and (3) both degrading mRNA and inhibiting mRNA translation. More and more pieces of animal and clinical evidence have indicated that miRNA is involved in the regulation of kidney development and diseases. For example, miR-26a is downregulated in renal cell carcinoma,^21^ and inhibition of miR-489 increases the apoptosis of renal tubular epithelial cells in ischemic kidney injury mice.^22^ The miR-30 family has five family members, including miR-30a, miR-30b, miR-30c, miR-30d and miR-30e, which possess common precursors with highly conserved sequences, indicating that they might regulate common target genes and be involved in similar renal physiological and pathophysiological events. In this study, we investigated the role of miR-30c in cisplatin-induced proximal tubular cell apoptosis, and our data revealed the direct target genes of miR-30c in this pathological process.

## Results

### Cisplatin exposure leads to the death of renal tubular epithelial cells and the reduction of miR-30a-e expression

**Figure 1:**
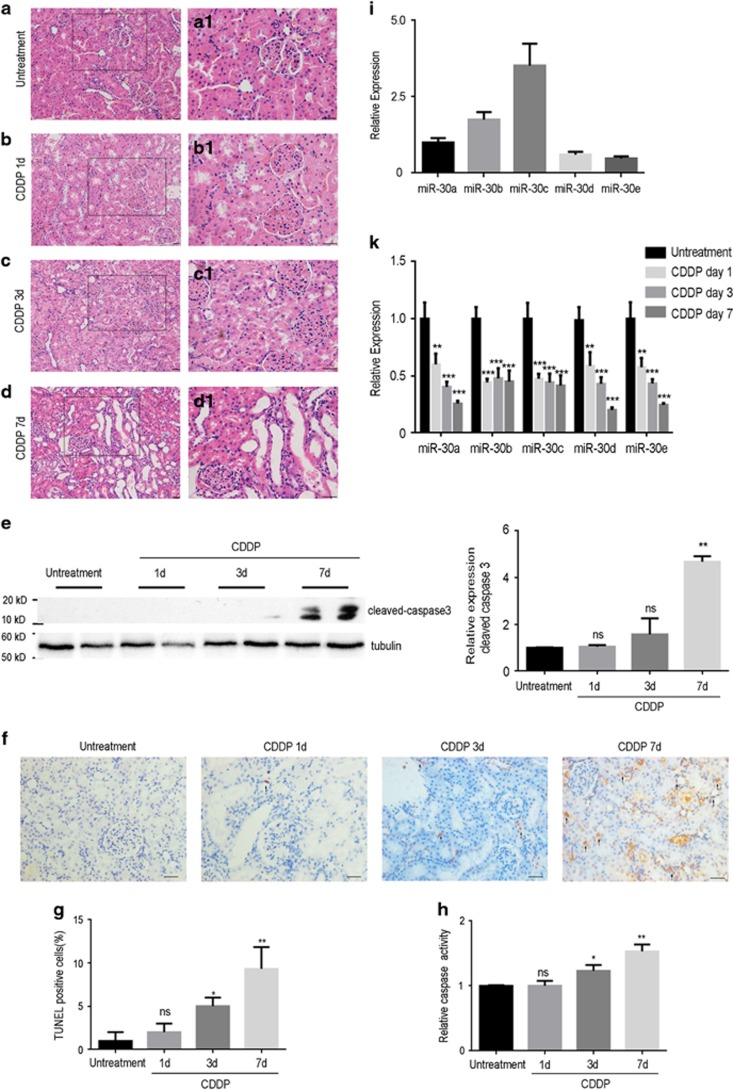
The histological characteristics and expression levels of miR-30a-e in rat renal proximal tubules treated with cisplatin. (**a**–**d**) Representative images of H&E-stained rat renal tubules are taken from the kidneys of untreated rats (**a**) and those treated with 10 mg/kg cisplatin for 1 day (**b**), 10 mg/kg cisplatin for 3 days (**c**) and 10 mg/kg cisplatin for 7 days (**d**). (**a1**–**d1**) The higher-magnification images focus on renal proximal tubules from **a** to **d**, respectively. (**e**) The western blotting data show the protein expression of cleaved caspase3 in rat renal tissue from the untreated and the 1-, 3- and 7-day cisplatin-treated groups. In addition, the bar chart shows the comparison of the relative expression of cleaved caspase3 (caspase3/tubulin) between the control group and the groups treated with cisplatin for different durations. (**f**) Representative TUNEL immunohistochemistry was implemented on kidney transverse sections from the untreated and the 1-, 3- and 7-day cisplatin-treated groups. Arrows indicate TUNEL-stained apoptotic nuclei of the tubular epithelial cells. (**g**) The bar chart shows the ratios of TUNEL-positive cells divided by total cells from 10 microscopic fields at × 400 magnification. (**h**) Caspase activity is measured by enzymatic assays using DEVD.AFC acts as the substrate. The bar chart shows caspase activity of cisplatin-treated groups over untreated group. (**i**) The bar chart shows the quantitative PCR data on the expression of miR-30a, miR-30b, miR-30c, miR-30d and miR-30e in the normal rat renal tissue. (**k**) The bar chart shows the quantitative PCR data in the expression of miR-30a, miR-30b, miR-30c, mR-30d and miR-30e in the rat renal tissue from the untreated and cisplatin-treated groups treated for 1, 3 and 7 days. Scale bars=200 *μ*m in **a**–**d** and **a1**–**d1**. Data represent the mean of three independent experiments±S.E.M. with *n*=5. *P<0.05; **P<0.01; ***P<0.001

An AKI animal model was established through intraperitoneal injection of cisplatin (10 mg/kg body weight) into 8-week-old Wistar rats at day 0. The untreated rats were intraperitoneally injected with the same amount of physiological saline. The rats were then killed to collect the kidneys on days 1, 3 and 7 after intraperitoneal injection. Hematoxylin & eosin (H&E) staining was performed on the transverse sections of the control and cisplatin-exposed rat kidneys at the assigned time (day 1, 3 or 7; Figures 1a–d). From the higher-magnification images of the renal tubules (Figures 1a1–d1), we could clearly see that the morphology of the proximal convoluted tubules appeared to be normal on day 1 of cisplatin injection compared to that in control (Figures 1a1 and b1). However, exposure to cisplatin for more than 3 days resulted in severe and widespread structural failure with dilatation, vacuolar degeneration, epithelial desquamation and intraluminal cast formation in the proximal convoluted tubules (Figures 1c1 and d1). Western blot data demonstrated that cleaved-caspase3 expression in renal tissue was slightly elevated on the day 3 of cisplatin exposure and was significantly upregulated on the day 7 (Figure 1e). As shown in Figures 1f–h, TUNEL (Figures 1f and g) and caspase activity assays (Figure 1h) demonstrated that exposing cisplatin for 3 days was enough to induce apoptosis of renal tubular cells, and the apoptosis significantly increased on the seventh day of cisplatin exposure. It suggests that cisplatin exposure indeed induces apoptosis in renal tubular epithelial cells within a short time period, which is consistent with previous studies.^23, 24^ Moreover, we detected the expression of the miR-30 family in renal tissue and found that miR-30c was the highest expressed miRNA among the five members (Figure 1i). Interestingly, we discovered that the expression levels of miR-30a, miR-30b, miR-30c, miR-30d and miR-30e were markedly inhibited by cisplatin exposure, especially that of miR-30b, miR-30c and miR-30e as the inhibition was significantly different from that on day 1 of cisplatin injection (Figure 1j).

**Figure 2:**
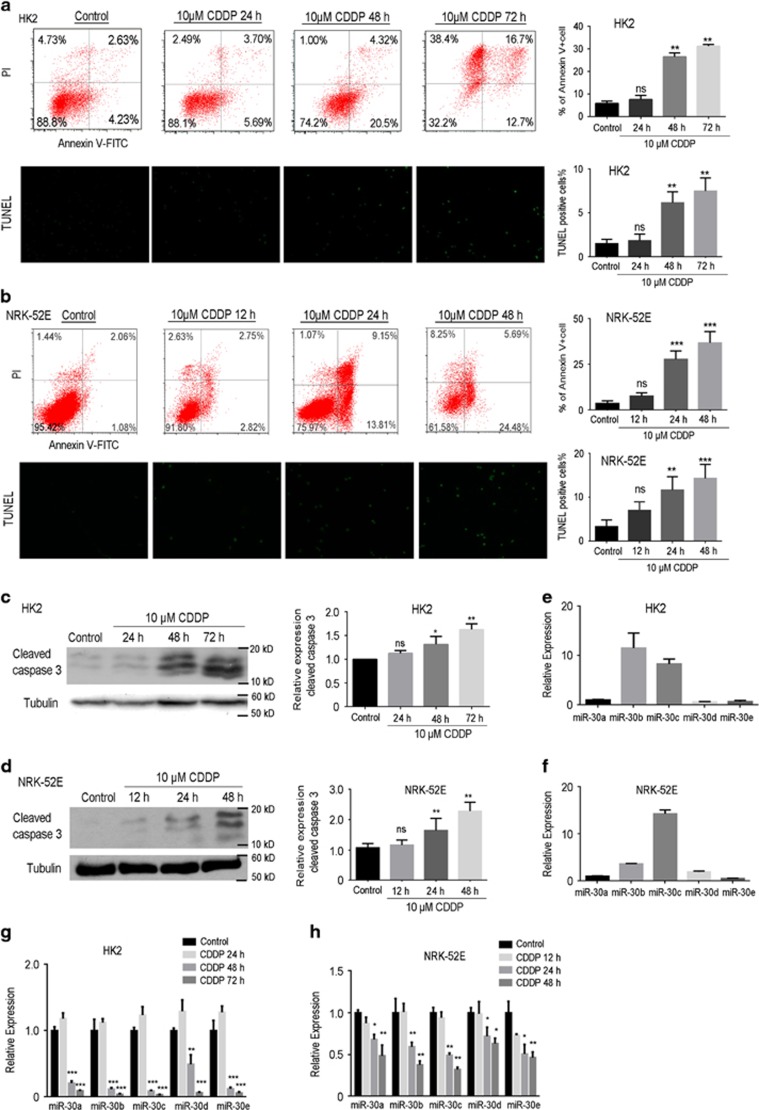
The assessment of apoptosis and the expression levels of miR-30a-e in HK-2 and NRK-52E cells treated with cisplatin. (**a** and **b**) HK-2 (**a**) or NRK-52E cells (**b**) were treated with 10 *μ*M cisplatin for different times. After treatment, HK-2 (**a**, upper panel) or NRK-52E (**b**, upper panel) cells were stained with Annexin V–FITC and PI for determining cell apoptosis using flow cytometry assay. The bar chart (right) shows the ratios of apoptosis cell numbers between the control and cisplatin-treated groups. The apoptosis of HK-2 (**a**, lower panel) and NRK-52E (**b**, lower panel) cells was also examined by TUNEL assay. The bar chart (right) shows the ratios of TUNEL-positive cell divided by total cells from 10 microscopic fields at × 400 magnification between the control and cisplatin-treated groups. (**c** and **d**) The western blotting data show the protein expression of cleaved caspase3 in HK-2 (**c**) and NRK-52E cells (**d**) from the control and cisplatin-treated groups. In addition, the bar charts (right) show the comparison of the relative expression of cleaved caspase3 (caspase3/tubulin) between the control and cisplatin-treated groups. (**e** and **f**) The bar chart shows the quantitative PCR data on the expression of miR-30a, miR-30b, miR-30c, miR-30d and miR-30e in the HK-2 (**e**) and NRK-52E cells (**f**). (**g** and **h**) The bar chart shows the quantitative PCR data on the expression of miR-30a, b, c, d and e in the HK-2 (**g**) or NRK-52E cells (**h**) of the control and cisplatin-treated groups. Data represent the mean of three independent experiments±S.E.M. with *n*=3. *P<0.05; **P<0.01; ***P<0.001

To strengthen the observation in renal tubules, we exposed HK-2 cells (an immortalized human proximal tubule epithelial cell line) and NRK-52E cells (a rat proximal tubule epithelial cell line) to 10 *μ*M cisplatin for 12, 24, 48 or 72 h *in vitro* (Figure 2). Then, flow cytometry was used to assess the apoptosis of HK-2 (Figure 2a) and NRK-52E (Figure 2b) cells following exposure to cisplatin. The results showed a significant increase in the percentage of Annexin V^+^ cells in the 24- or 48 h-treated group compared to that in the control group. Again, the data from western blot analysis showed a significant increase in cleaved-caspase3 expression in the 48 h-treated HK-2 cells (Figure 2c) and 24 h-treated NRK-52E cells (Figure 2d), indicating that there is no doubt that cisplatin exposure results in apoptosis of renal tubular epithelial cells. Likewise, we revealed that the expression levels of miR-30b and miR-30c were higher than that of the other three members in HK-2 (Figure 2e) and NRK-52E (Figure 2f) cells. Furthermore, we also demonstrated that miR-30a-e expression was markedly inhibited in HK-2 (Figure 2g) and NRK-52E (Figure 2h) cells exposed to cisplatin, further confirming the observation in renal tissue in Figure 1.

### Manipulating miR-30c expression alters the effect of cisplatin-induced apoptosis in proximal tubule epithelial cells

As shown on Figures 1 and 2, miR-30b and miR-30c were expressed stronger than miR-30a, miR-30d and miR-30e in renal tubular cells and tissues. The expressions of miR-30a-e were downregulated in cisplatin-treated renal tissue or renal tubular cell. Of course, all of miRNA-30a-e might have role on the regulation of apoptosis. However, even slight variation of the highly abundant miRNA could lead to the significant alteration of target gene expressions. Therefore, the highly abundant miRNA probably has more important role than less-abundant miRNA on maintaining cellular physiological functions.^25^ Furthermore, previous studies demonstrated that the dysregulation of miR-30c would cause several serious kidney diseases such as diabetic nephropathy,^26^ renal fibrosis,^27^ ischemia-reperfusion-induced kidney injury^28^ and contrast-induced AKI.^29^ Hence, miR-30c was chosen for the following functional experiments in this study.

**Figure 3:**
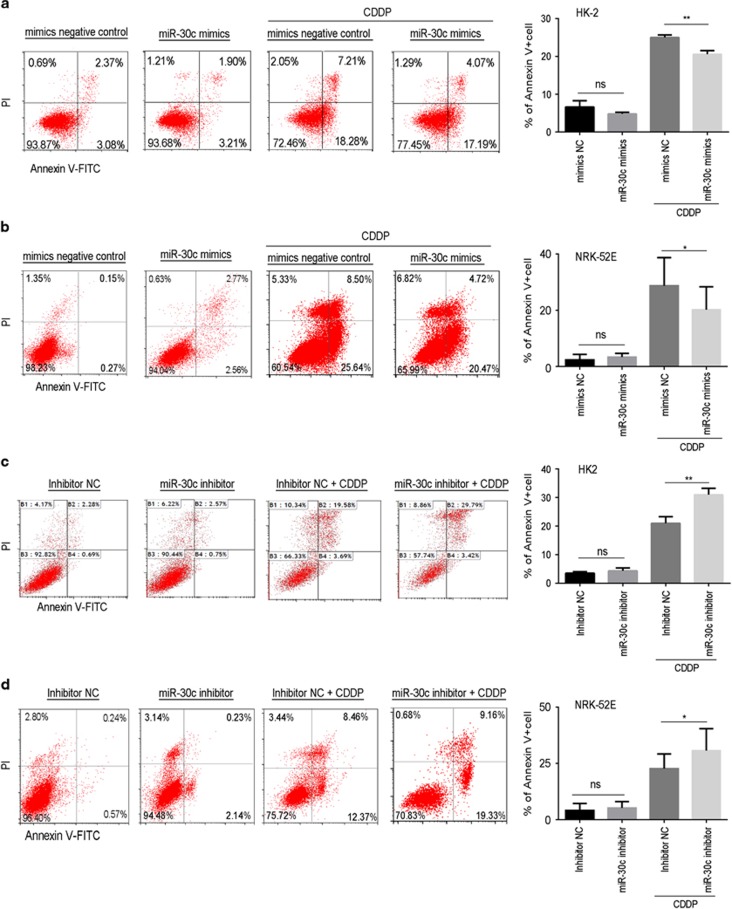
The assessment of apoptosis in HK-2 and NRK-52E cells treated with cisplatin and inhibitors or mimics of miR-30 c. (**a** and **b**) HK-2 cells (**a**) or NRK-52E cells (**b**) were treated with mimics of miR-30c in the absence/presence of 10 *μ*M cisplatin for 24 or 48 h before flow cytometry analysis (left). The bar chart (right) shows the ratios of apoptosis cell numbers between the groups treated with the negative control mimic, miR-30c mimic, negative control mimic+cisplatin or miR-30c mimic+cisplatin. (**c** and **d**) HK-2 cells (**c**) or NRK-52E cells (**d**) were treated with inhibitors of miR-30c in the absence/presence of 10 *μ*M cisplatin for 24 or 48 h before flow cytometry analysis (left). The bar chart (right) shows the ratios of apoptosis cell numbers between the groups treated with the negative control inhibitor, miR-30c inhibitor, negative control inhibitor+cisplatin or miR-30c inhibitor+cisplatin. Data represent the mean of three independent experiments±S.E.M. with *n*=3. *P<0.05; **P<0.01

To investigate whether miR-30c is involved in regulating cisplatin-induced apoptosis, we used the transfection of either the miR-30c mimic (upregulation) or miR-30c inhibitor (downregulation) in HK-2 cells and NRK-52E cells (Figure 3). Flow cytometry was used to determine cell apoptosis following the manipulation of miR-30c expression in HK-2 (Figure 3a) and NRK-52E (Figure 3b) cells. In the presence of the miR-30c mimic (upregulation), we could see that the miR-30c mimic alone did not affect apoptosis, but upregulation of miR-30c with the mimic significantly decreased the cisplatin-induced elevated apoptosis. In the presence of the miR-30c inhibitor, we first displayed that there was no difference in apoptosis between treatment with the miR-30c inhibitor only and the negative control inhibitor; however, downregulation of miR-30c with the inhibitor significantly aggravated cisplatin-induced cell apoptosis in HK-2 and NRK-52E cells as shown in Figures 3c and d. These results suggest that miR-30c might be involved in regulating cisplatin-induced cell apoptosis.

### *Bnip3L* and *Hspa5* could be the target genes of miR-30c in cisplatin-induced apoptosis in renal tubular epithelial cells

**Figure 4:**
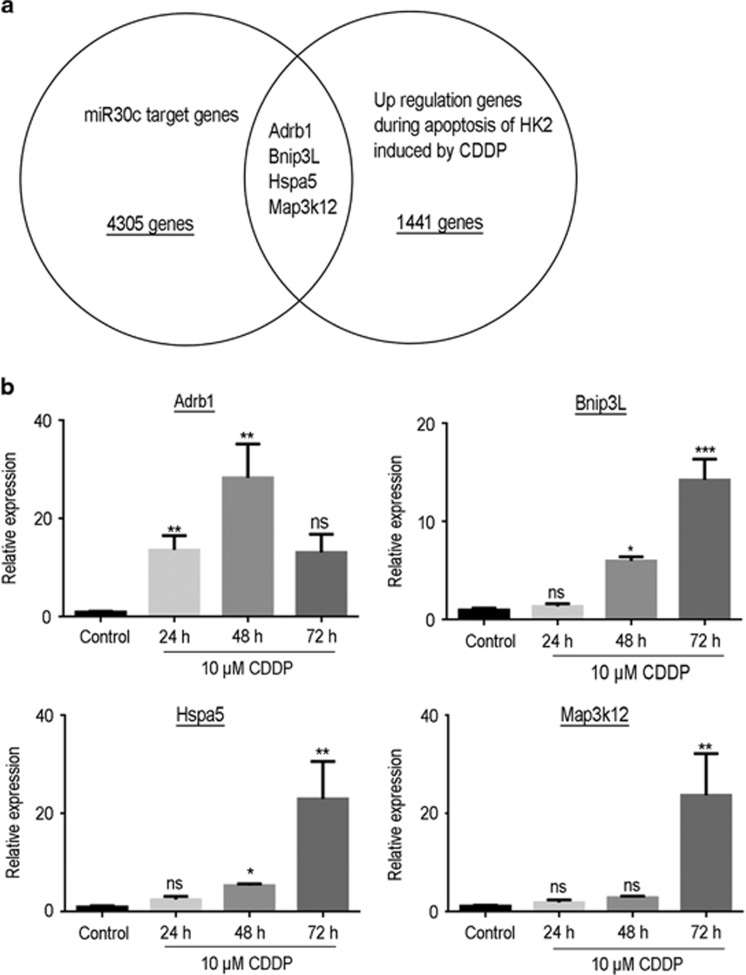

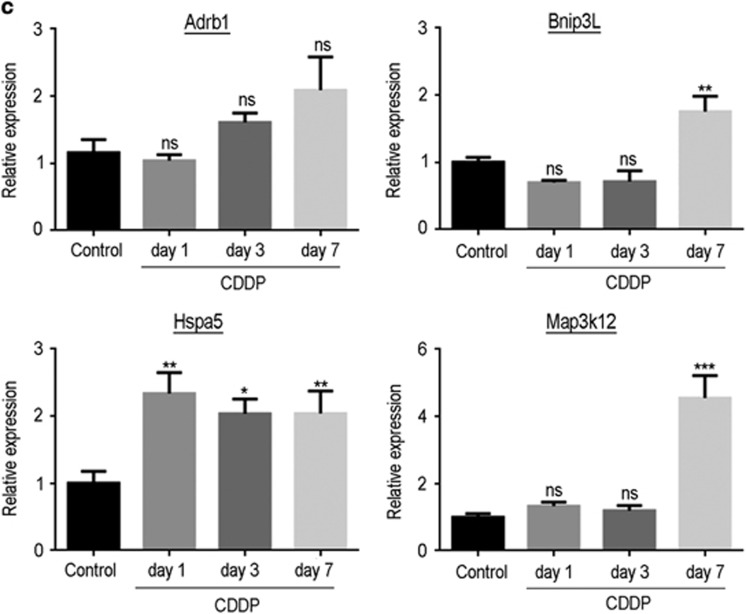
Bioinformatics predicted the four potential target genes in cisplatin-induced renal tubule injury. (**a**) TargetScan was used to predict the potential target genes of miR-30c, and four target genes (*Adrb1*, *Bnip3L*, *Hspa5* and *MAP3K12*) were found to be upregulated in cisplatin-treated HK-2 cells according to the CEO database (GSE69644). (**b**) The bar chart showing the quantitative PCR data on the mRNA expression levels of *Adrb1*, *Bnip3L*, *Hspa5* and *MAP3K12* in HK-2 cells of control and 48 and 72 h cisplatin-treated groups. (**c**) The bar chart showing the quantitative PCR data on the mRNA expression levels of *Adrb1*, *Bnip3L*, *Hspa5* and *MAP3K12* in the rat renal tissue of the control and the 1-, 3- and 7-day cisplatin-treated groups. Data represent the mean of three independent experiments±S.E.M. with *n*=3. *P<0.05; **P<0.01; ***P<0.001

To explore the target genes of miR-30c, we used bioinformatics tools, including TargetScan. The intersected genes that were predicted from the three software programs were deemed to be possible target genes (*n*=4305). Furthermore, we looked for the elevated genes (*n*=1441) expressed in renal tubular epithelial cells in both cisplatin-induced kidney injury and the HK-2 apoptosis models using the GEO database (GSE69644).^30^ We preliminarily chose *Adrb1*, *Bnip3L*, *Hspa5* and *MAP3K12* through comparing the elevated genes and the miR-30c target gene prediction (Figure 4a).

To further confirm which genes could be the target genes for miR-30c, we determined the expression levels of *Adrb1*, *Bnip3L*, *Hspa5* and *MAP3K12* in renal tubular epithelial cells and HK-2 cells exposed to cisplatin (Figures 4b and c). When the cells were treated with cisplatin, we found that cisplatin exposure significantly increased the expression of *Bnip3L* and *Hspa5* but not the expression of *Adrb1* and *MAP3K12* in renal tubular epithelial cells (Figure 4b). Likewise, we also found that the expression levels of *Bnip3L* and *Hspa5* were elevated by cisplatin exposure in renal tissue (Figure 4c), but not much of a significant change was found in the expression of *Adrb1* and *MAP3K12*, indicating that *Bnip3L* and *Hspa5* were more likely to be the target genes for miR-30c.

**Figure 5:**
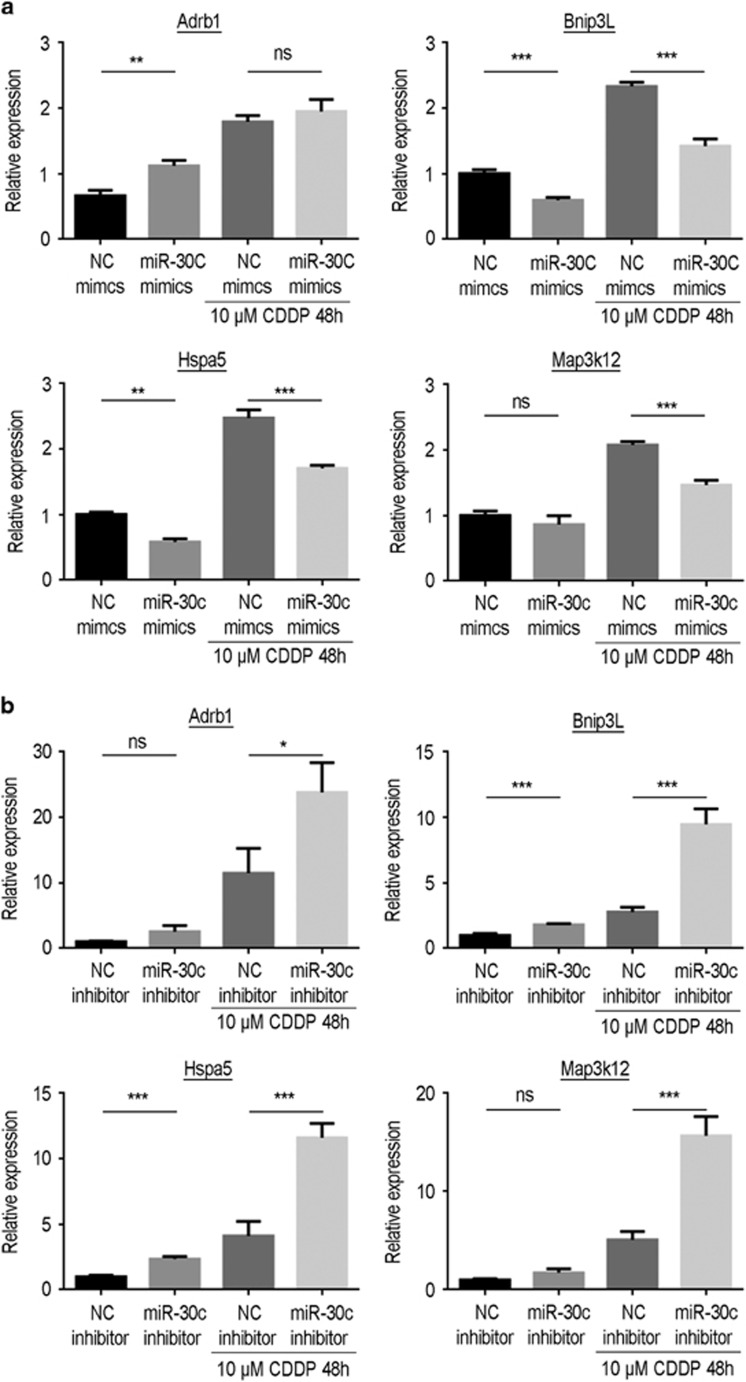
The expression levels of *Bnip3L* and *Hspa5* in HK-2 cells after manipulating miR-30c expression in the presence of cisplatin. (**a**) HK-2 cells were treated with mimics of miR-30c in the absence/presence of 10 *μ*M cisplatin for 48 h. The bar charts show the quantitative PCR data on the expression levels of *Bnip3L* and *Hspa5* in the negative control mimic, miR-30c mimic, negative control mimic+cisplatin and miR-30c mimic+cisplatin groups. (**b**) HK-2 cells were treated with an inhibitor of miR-30c in the absence/presence of 10 *μ*M cisplatin for 48 h. The bar charts show the quantitative PCR data on the expression levels of *Bnip3L* and *Hspa5* in the negative control inhibitor, miR-30c inhibitor, negative control inhibitor+cisplatin and miR-30c inhibitor+cisplatin groups. Data represent the mean of three independent experiments±S.E.M. with *n*=3. *P<0.05; **P<0.01; ***P<0.001

To be on the safe side, we determined the expression levels of *Adrb1*, *Bnip3L*, *Hspa5* and *MAP3K12* in HK-2 cells following either downregulation or upregulation of miR-30c (Figure 5). As shown in Figure 5a, only the expression levels of *Bnip3L* and *Hspa5* were reduced when miR-30c was upregulated with the miR-30c mimic. Furthermore, the elevated expression of both *Bnip3L* and *Hspa5* induced by cisplatin exposure was significantly suppressed by overexpression of miR-30c. In addition, when miR-30c was downregulated with the miR-30c inhibitor, we distinctly found that the expression of *Bnip3L* and *Hspa5* was elevated. Moreover, the cisplatin-induced elevated expression of *Bnip3L* and *Hspa5* was increased in the miR-30c inhibitor groups (Figure 5b). Taken together, we speculate that *Bnip3L* and *Hspa5* could be the target genes in the cisplatin-induced apoptosis of renal tubular epithelial cells.

**Figure 6:**
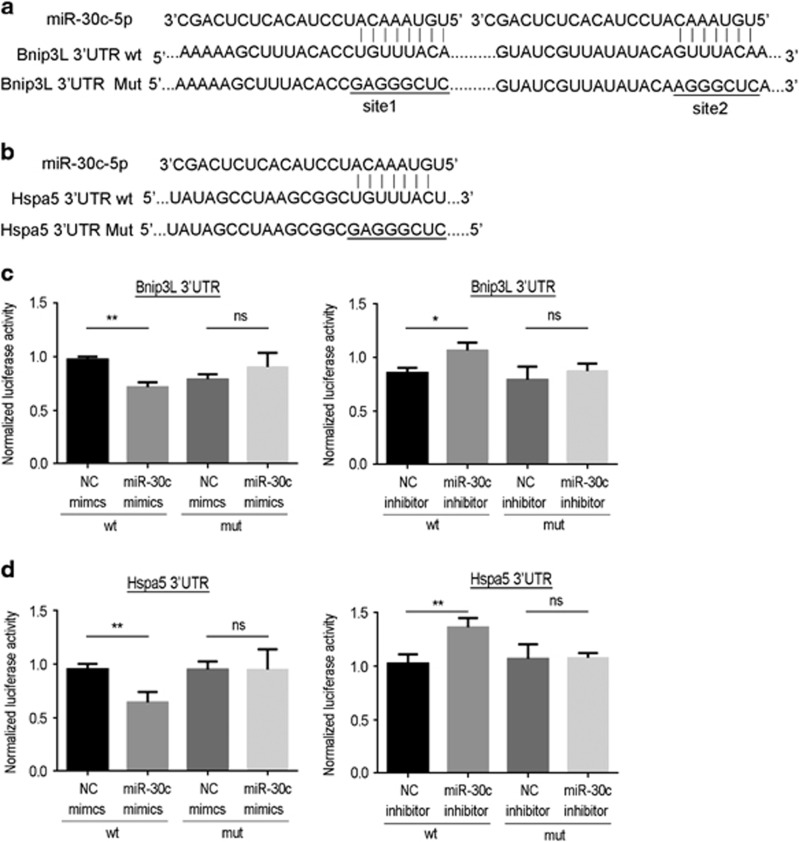
MiR-30c directly targets *Bnip3L* and *Hspa5*. (**a** and **b**) The sequence alignment between miR-30c and the predicted binding sites or mutational site in the 3′-UTR of *Bnip3L* and *Hspa5*. (**c** and **d**) Firefly luciferase reporters containing either wild-type (WT) or mutant (Mut) *Bnip3L* or *Hspa5* 3′-UTR were co-transfected into HK-2 cells with the miR-30c mimic, negative control (NC), miR-30c inhibitor or NC inhibitor. Forty-eight hours after transfection, cells were assayed for firefly luciferase and Renilla luciferase assays. Data represent the mean of three independent experiments±S.E.M. with *n*=4. *P<0.05; **P<0.01

Then, we further performed luciferase reporter assays using reporters carrying either the wild-type 3′-untranslated region (UTR) of human *Bnip3L* and *Hspa5* or mutant nucleotides swapped in the region corresponding to the miR-30c ‘seed’ (Figures 6a and b). When HK-2 cells were co-transfected with the synthetic miR-30c mimic, the wild-type reporter exhibited reduced luciferase activity, but the mutant did not. Moreover, the wild-type reporter but not the mutant yielded a higher luciferase activity in miR-30c inhibitor-transfected cells (Figures 6c and d). These results indicate that *Bnip3L* and *Hspa5* genes function as the direct targets of miR-30c during the proximal tubular cell apoptosis caused by cisplatin.

**Figure 7:**
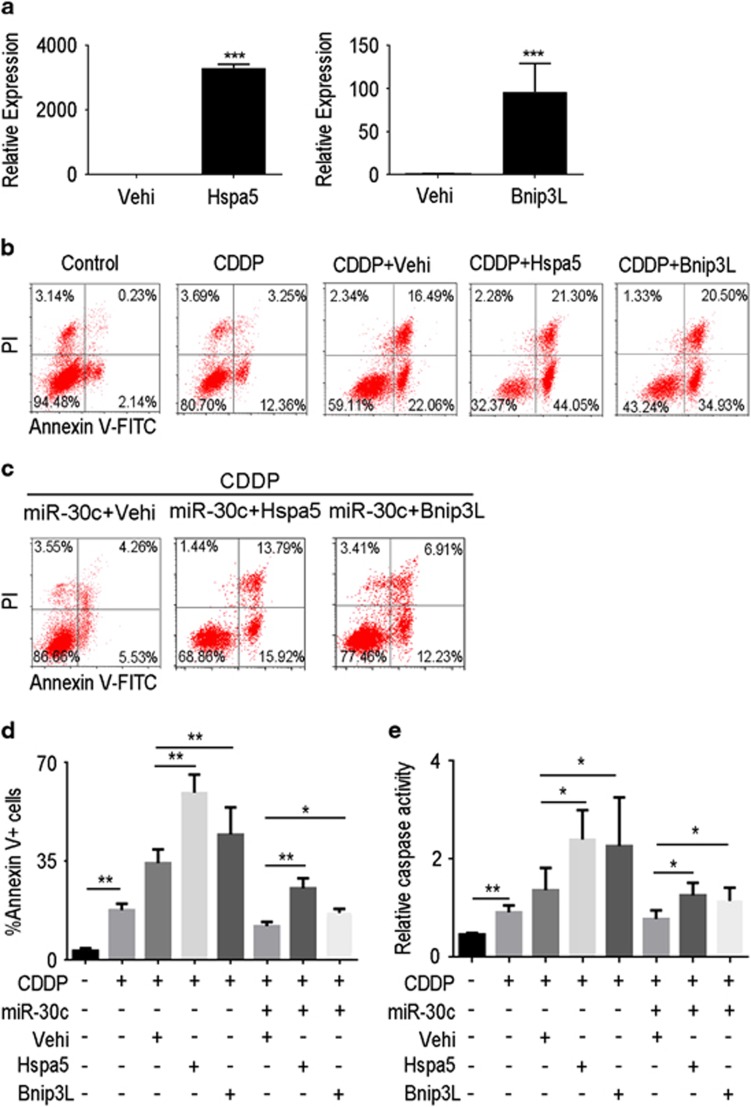
*Hspa5* or *Bnip3L* overexpression abolished miR-30c effects on cisplatin-induced apoptosis. (**a**) After transfected with empty vector (vehicle, vehi), *Hspa5* and *Bnip3L* plasmid, mRNA levels of *Hspa5* or *Bnip3L* in HK-2 cells were detected by qRT-PCR. (**b**) HK-2 cells were transfected with empty vector, *Hspa5* and *Bnip3L* plasmid DNA in the presence of 20 *μ*M cisplatin for 24 h before Annexin V flow cytometry analysis. (**c**) HK-2 cells were transfected with miR-30c mimics and plasmid DNA, and then incubated in the presence of 20 *μ*M cisplatin for 24 h before Annexin V flow cytometry analysis. (**d**) The bar chart shows the ratios of apoptosis cell numbers between the groups. (**e**) The bar chart showing caspase activities of different treatment group by enzymatic assays using DEVD.AFC acts as the substrate. Data represent the mean of three independent experiments±S.E.M. with *n*=4. *P<0.05; **P<0.01; ***P<0.001

We further studied the role of *Hspa5* and *Bnip3L* in proximal tubular cell apoptosis by transferring *Bnip3L* or *Hspa5* plasmid DNA into the HK-2 cells. As shown in Figure 7a, *Bnip3L* or *Hspa5* plasmid DNA transfection was able to efficiently upregulate *Bnip3L* or *Hspa5* expression, respectively. And we found that the overexpression of *Hspa5* or *Bnip3L* resulted in significant increase on cell apoptosis induced by cisplatin (Figure 7b). To assess whether or not *Bnip3L* or *Hspa5* contributed proximal tubular cell apoptosis induced by cisplatin, we overexpressed Bnip3L or Hspa5 in HK-2 cells along with miR-30c overexpression and cisplatin treatment. As shown on Figures 7c and d, *Bnip3L* or *Hspa5* overexpression abolished miR-30c effects on the cisplatin-induced apoptosis. The result of caspase activity assay (Figure 7e) was consistent with Annexin V/PI assay. All these data further confirm that *Bnip3L* or *Hspa5* serve as the target genes of miR-30c and mediated renal tubular cell apoptosis induced by cisplatin.

## Discussion

Cisplatin is most commonly used as a therapeutic agent in treating tumors, and its nephrotoxicity markedly limits its clinical application. Therefore, studying the mechanism of cisplatin-induced nephrotoxicity is undoubtedly important and is the basis for exploring novel strategies to minimize the side effects. MiRNAs are a group of endogenously synthesized non-coding RNAs that have been considered to be important regulators of physiological and pathophysiological conditions such as development and tumorigenesis. The regulatory role of microRNAs in various physiological and pathological processes, including AKI, has gradually become known. Zhu *et al.*^31^ reported that the CDDP-induced apoptosis of tubular epithelial cells was modulated by the suppression of Bcl-2 expression by miR-181a. Bhatt *et al.*^32^ reported that miR-34a might have a cytoprotective role for renal tubular cells through p53 during cisplatin-induced nephrotoxicity. Lee *et al.*^33^ showed that cisplatin treatment mostly downregulated miR-122, whereas it upregulated miR-34a expression using microarray analyses in mouse kidneys injured by treatment with cisplatin, indicating that both miR-34a and miR-122 are involved in the molecular biological mechanism of cisplatin-induced nephrotoxicity.

In this study, we focused on the role of the miR-30 family in the protection of renal tubular cells from the injury induced by cisplatin. The reason for focusing on this family is that the miR-30 family is the highest abundant miRNA family in renal tubular epithelial cells according to the results of our previous gene microarray.^34^ Here we revealed that all of the miR-30 miRNAs, including miR-30a, miR-30b, miR-30c, miR-30d and miR-30e, were downregulated in both renal tubular epithelial cells and HK-2 cells when cell apoptosis in renal tubules was induced by cisplatin exposure (Figures 1 and 2). Furthermore, the cisplatin-induced apoptosis in HK-2 and NRK-52E cells was affected by changes in miR-30c. Cell apoptosis was attenuated when miR-30c was activated, while miR-30c inhibition caused the aggravation of cell apoptosis (Figure 3). Although the *in vivo* evidence about the role of miR-30c on cisplatin-induced renal tubular cell apoptosis lacks, our current experimental results in this study are generally consistent with the observation in which podocyte apoptosis induced by either TGF-beta or puromycin aminonucleoside treatment was ameliorated by exogenously expressing miR-30 and aggravated by the knockdown of miR-30.^35^ In addition, miR-30a-5p has been identified to exert a role as a tumor-suppressing microRNA in colon cancer cells through modulating the expression of DTL, which is overexpressed to arrest the cell cycle at the G(1) phase and induce apoptosis in colorectal cancer.^36^ Roca-Alonso *et al.*^37^ reported that miR-30 overexpression protects cardiac cells from doxorubicin-induced apoptosis. All of these reports and our results reveal that the miR-30 family indeed has a regulatory role in the modulation of the cell cycle and cell apoptosis in a variety of pathophysiological processes.

To elucidate the target genes of miR-30 in cisplatin-induced cell apoptosis, we used bioinformatics tools to predict potential potent target genes. By comparing the upregulated genes in the cisplatin-induced kidney injury models, we chose putative genes, including *Adrb1*, *Bnip3L*, *Hspa5* and *MAP3K12*, as possible target genes of miR-30 s as they were in the intersection of both database queries (Figure 4a). Further assessment of these four genes (*Adrb1*, *Bnip3L*, *Hspa5* and *MAP3K12*) was implemented to determine the effect of cell apoptosis induced by cisplatin in renal tubular epithelial cells and HK-2 cells, and the results indicated that *Bnip3L* and *Hspa5* were more likely the target genes of miR-30c as the expression levels of both genes showed significant alterations in the presence of cisplatin induction (Figures 4b and c). *Bnip3L* (BCL2/adenovirus E1B 19 kDa protein-interacting protein 3-like) is one member of the BCL2 family, and it interacts with the E1B 19 kDa protein to regulate cell death as an apoptosis protector. As a functional homolog of BNIP3, *Bnip3L* may have a role in tumor suppression, working with BNIP3 simultaneously.^38^ Guo *et al.*^38^ demonstrated that miR-30e, as a member of the miR-30 family, protected against aldosterone-induced podocyte apoptosis and mitochondrial dysfunction by directly targeting *Bnip3L*. As a member of the heat shock protein 70 family, the *Hspa5* (heat shock 70 kDa protein 5) gene appears in the lumen of the endoplasmic reticulum, and its cellular function is implicated in the assembly of proteins in the endoplasmic reticulum through interacting with many endoplasmic reticulum proteins.^39^ Wang *et al.*^40^ reported that miR-30a inhibition prevented neural ischemic injury through upregulating *Hspa5* protein expression, in which the mechanism underlying the *Hspa5*-mediated neuroprotection was a reduction in endoplasmic reticulum stress-induced apoptosis, suggesting that *Bnip3L* and *Hspa5* have been known to be modulators of the process of cell apoptosis. Finally, upregulation of miR-30c was shown to alleviate the cisplatin-induced high expression levels of *Bnip3L* and *Hspa5*, while downregulation of miR-30c aggravated the cisplatin-induced high expression levels of *Bnip3L* and *Hspa5* (Figure 5). The results from previous studies further confirmed our hypothesis that *Bnip3L* and *Hspa5* are target genes of miR-30c based on our current experimental results.

In sum, the nephrotoxicity of cisplatin has been very well known and markedly limits the application of cisplatin as a commonly used anticancer drug. In this study, we first revealed that the expression of miR-30a-e was reduced in cisplatin-induced renal tubular cells apoptosis *in vitro* and *in vivo*. Manipulating miR-30c expression in HK-2 and NRK-52E cells directly altered the effect of cisplatin-induced cell apoptosis. Using bioinformatics tools, we predicted putative genes, including *Adrb1*, *Bnip3L*, *Hspa5* and *MAP3K12*, to be the target genes of miR-30. Subsequently, *Bnip3L* and *Hspa5* were confirmed to more likely be the target genes of miR-30c in cisplatin-induced injury of renal tubules. There is no doubt that our current study on miR-30c function may markedly contribute to the goal to minimize cisplatin-induced nephrotoxicity, although the precise molecular biological mechanism and *in vivo* experimental evidence how miR-30c mediates apoptosis of renal tubular cell must still be addressed in the future.

## Materials and methods

### Experimental animals

The Wistar rats (average weight 260 g) in this study were obtained from the Laboratory Animal Centre of South Medical University (Guangzhou, China). The rats were housed in an alternate light–dark cycle (every 12 h) room with a temperature of 22±2 °C and a relative humidity of 50–60%. In addition, the rats were fed with a complete formula food and allowed water *ad libitum*. Eight-week-old female rats were used for this study after 10 days of adaption. Twenty-eight rats were randomly divided into four groups (seven rats for each group) to implement this experiment and were injected with either physiological saline (control) or cisplatin (Sigma, St. Louis, MO, USA) dissolved in 0.01 M citrate buffer at a pH of 4.5 and a dose of 10 mg/kg body weight for 1, 3 or 7 consecutive days (day 1, 3 or 7) before killing the animal. All processes involving animal treatment in this study were in accordance with the procedures of the Ethical Committee for Animal Experimentation, Jinan University.

### Renal morphological analysis

The rat kidneys were collected at the assigned time. Kidneys were photographed and fixed in 4% paraformaldehyde (PFA) and then dehydrated, embedded in paraffin wax and serially sectioned at 2 *μ*m thickness. For histology, the sections were de-waxed in xylene, rehydrated and stained with H&E stain. The sections were photographed using a fluorescence microscope (Eclipse Ti-E, Nikon, Tokyo, Japan) linked to NIS-Elements F3.2 software (Nikon,Tokyo, Japan). Representative renal tubules were determined and chosen through carefully viewing the renal transverse sections; a minimum of five images randomly chosen from five samples were assayed per group.

### Cell culture and gene expression interference

HK-2 (an immortalized human proximal tubule epithelial cell line) and NRK-52E cells (a rat proximal tubule epithelial cell line) were purchased from American Type Culture Collection (Manassas, VA, USA). The cells were cultured in a humidified incubator with 5% CO_2_ at 37 °C in six-well plates (1 × 10^6^ cells/ml) containing DMEM/F-12 medium (Gibco, Gaithersburg, MD, USA) supplemented with 10% fetal bovine serum (Gibco) and exposed to 10 *μ*M cisplatin (Sigma) for the assigned duration. Cells were plated to 50–70% confluence, and the treatment was subsequently added to the cells. The treated cells were collected at the assigned time.

### Western blotting

Western blotting was performed in accordance with a standard procedure using a polyclonal antibody that specifically recognized cleaved caspase3 (1:1000, Cell Signaling Technology, Danvers, MA, USA).^41^ The collected renal tissue, HK-2 cells and NRK-52E cells were frozen in liquid nitrogen and kept at −80 °C. Protein from the renal tissue, HK-2 cells and NRK-52E cells were isolated from tissue homogenates or cell lysates using a radioimmunoprecipitation assay (Sigma) buffer supplemented with protease and phosphatase inhibitors. Protein concentrations were quantified with the BCA assay. The extracted protein was separated by 10% SDS-PAGE and transferred onto a polyvinylidene difluoride membrane (Millipore, Temecula, CA, USA). The membrane was blocked with 5% nonfat milk and then incubated with the cleaved caspase3 antibody (1:1000, Cell Signaling Technology) in TBS buffer at 4 °C overnight. The loading control was a tubulin antibody (1:3000, Proteintech, Rosemont, IL, USA). After incubation with the secondary antibody, either HRP goat anti-rabbit IgG (1:3000, EarthOx, Millbrae, CA, USA) or HRP goat anti-mouse IgG (1:3000, EarthOx), the blots were developed with SuperSignal West Femto Chemiluminescent Substrate (ThermoFisher, Rockford, IL, USA), Gel Doc XR+ System (BIO-RAD, Hercules, CA, USA). ImageJ software (NIH, Bethesda, MD, USA) was used to capture the chemiluminescent signals and analyze the data.

### Examination of apoptosis

#### Fluorogenic caspase activity assay

DEVD.AFC, a fluorogenic peptide substrate of caspases, was used to measure the enzymatic activity of caspases in cell lysatesas, as described in publications.^41, 42^ A total of 3–5 × 10^6^ cells were rinsed with ice-cold phosphate-buffered saline (PBS) and subsequently cell lysis buffer was added (Cell Signaling Technology). For kidney specimens, 50–100 mg tissue were cut off completely with scissors and subsequently cell lysis buffer was added. Caspase activity assay (Cell Signaling Technology) was performed according to the manufacturer’s instructions.^41^ After 1 h of reaction at 37 °C, fluorescent intensity was measured with excitation wavelength at 405 nm and an emission wavelength of 530 nm and expressed in relative fluorescence units.

#### Annexin V-fluorescein isothiocyanate/propidium iodide staining

Annexin V-fluorescein isothiocyanate/propidium iodide staining was performed using a kit from BD Pharmingen (San Jose, CA, USA).^43^ Briefly, cells were detached by trypsinization and collected by centrifugation at 1000 × *g* for 5 min. The cells were then resuspended in binding buffer at a density of 1–2 × 10^6^ cells/ml. The 100 *μ*l single-cell suspension (1–2 × 10^5^ cells) was incubated with 5 *μ*l Annexin V-fluorescein isothiocyanate and 5 *μ*l propidium iodide for 15 min at room temperature. Finally, the mixture was diluted with 500 *μ*l binding buffer and analyzed with a FACSCalibur flow cytometer (NovoCyte, ACEA Biosciences, San Diego, CA, USA). For each sample, total of 10 000 events were counted.

#### TUNEL staining

TUNEL staining was conducted as previously.^41, 43^ Cells were seeded on glass coverslips in six-well plates and treated as mentioned above. After removal of culture medium, cells were rinsed with pre-chilled PBS thrice and fixed in 4% PFA for 20 min. Apoptotic cells were detected with *In Situ* Cell Death Detection Kit (Roche Diagnostics, Indianapolis, IN, USA) according to the manufacturer’s instructions. Cells were co-stained with DAPI and visualized with a fluorescence microscopy. For formalin-fixed paraffin-embedded tissue, the slides were prepared according to conventional methods. And then the tissue was washed in 0.1 M PBS, pH 7.4, for 5 min, treated with 0.2% H_2_O_2_ for 20 min, rinsed in 0.1 M PBS for 5 min and incubated in TUNEL reaction mixture from the *In Situ* Cell Death Detection Kit, POD for 1 h at 37C. The tissue was further rinsed in 0.1 M PBS three times for 5 min and incubated in converter-peroxidase (POD) from the *In Situ* Cell Death Detection Kit for 30 min at 37C, rinsed in 0.1 M PBS three times for 5 min and color-developed with diaminobenzidine POD substrate.

### RNA isolation and real-time RT-PCR

Total RNA from cultured cells or renal cortex tissues were extracted by Trizol reagent according to the protocol recommended by the manufacturer (Invitrogen, Carlsbad, CA, USA). Concentration and purity were measured using NanoDrop 2000 (Thermo Scientific, Rockford, IL, USA). The RNA integrity values ranged between 1.8 and 2.7. Equal amounts (2.0 *μ*g) of DNA-free total RNA from each sample were converted to cDNA using the PrimeScript RT reagent kit (Takara, Tokyo, Japan). Real-time PCR was carried out using SYBR Premix ExTaq (Takara) according to the manufacturer’s instructions. The sets of primers used for RT-PCR are provided in Supplementary Figure S1. PCR was performed in a BIO-RAD S1000 Thermal cycler (BIO-RAD). The miR-30 family and U6 qPCR were carried out using All-in-One qPCR mix and validated primers purchased from GeneCopoeia Corporation (Rockville, MD, USA). The results were reported from triplicate independent experiments.

### Plasmid, miRNA mimic and miRNA inhibitor transfection

The *Bnip3L* and *Hspa5* expression vector was generated by inserting its open reading frame into the pEGFP-C3 vector. All of mimic negative control, miR-30c mimic, miRNA inhibitor negative control and miR-30c inhibitor were purchased from Ribobio (Guangzhou, China). The cells were transfected with the plasmid, miRNA mimic or miRNA inhibitor using FuGENE HD (Roche, Basel, Switzerland) according to the manufacturer’s instructions. After transfected for 24 h, cells were used to the following experiments.

### Luciferase reporter assay

The wild-type 3′-UTR fragment of human *Bnip3L* and *Hspa5* that contained the putative binding site for miR-30c was obtained by PCR. The amplified fragment was inserted into the psiCHECK-2 vector (Promega, Madison, WI, USA), sequenced and then cloned downstream of the firefly luciferase coding region in psiCHECK-2. The mutant 3′-UTR fragment that carried a mutated putative binding site for miR-30c was generated by site-specific mutagenesis using a mutated PCR primer in the psiCHECK-2 vector. To perform the luciferase reporter assay, HK-2 cells grown in a 24-well plate were co-transfected with 0.5 *μ*g of recombinant psiCHECK-2, 1 ng of control plasmid (GenePharma, Shanghai, China) and 50 nM miRNA mimic or 100 nM miRNA inhibitor using Lipofectamine 2000 (Invitrogen, Carlsbad, CA, USA) and incubated for 24 h. Luciferase assays were performed 48 h after transfection using the Dual-Luciferase Reporter Assay System (Promega) on a GloMax 96 Microplate Luminometer (Promega). Renilla luciferase activity was normalized to the firefly luciferase expression for each sample.

### Data analysis

Data analyses and construction of statistical charts were performed using the GraphPad Prism 6 software package (GraphPad Software, La Jolla, CA, USA). The results are presented as the mean value (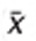
±S.E.M.). All data were analyzed using ANOVA or *t*-test, which were used to establish whether there was any difference between the control and experimental data. *P*<0.05 was considered to be significantly different.

## Publisher’s Note

Springer Nature remains neutral with regard to jurisdictional claims in published maps and institutional affiliations.
