# Supplementary material for: MiR-30c regulates cisplatin-induced apoptosis of renal tubular epithelial cells by targeting Bnip3L and Hspa5
Source: Cell Death Dis. 2017 Aug 10;8(8):e2987–. doi: 10.1038/cddis.2017.377 (PMC5596565; doi:10.1038/cddis.2017.377)
Supplement: Supplementary Information [file cddis2017377x1.pdf]

Primer sequence

Primer Name	sequence
Homo	
ADRB1	Forward: 5'-AGGGGAACGAGGAGATCTGT-3'
	Reverse: 5'-CAGACGAGGATTGTGGGCTT-3'
BNIP3L	Forward: 5'-CAATGTCGTCCCACCTAGTCG-3'
	Reverse: 5'-TAGCTCCACCCAGGAACTGTTG-3'
MAP3K12	Forward: 5'-CCTCAAAGAAACCGACATCAAGC-3'
	Reverse: 5'-GGATGCAGTAGCAGGGAGC-3'
HSPA5	Forward: 5'-CCCGAGAACACGGTCTTTGA-3'
	Reverse: 5'-TTCAACCACCTTGAACGGCA-3'
ACTB (actin, beta)	Forward: 5'- ACGTGGACATCCGCAAAG-3'
	Reverse: 5'- GACTCGTCATACTCCTGCTTG-3'
Rat	
Adrb1	Forward: 5'- ATGGGTGTGTTCACGCTCTG-3'
	Reverse: 5'- CAGCCAGTTGAAGAAGACGA-3'
Bnip3L	Forward: 5'- AGGCTAACCTGCAGCACAGT-3'
	Reverse: 5'- CACTGCCGATGAAACTGCTA-3'
Map3k12	Forward: 5'- CACTATGAGCGCAAGTTGGA-3'
	Reverse: 5'- GAGGGCATTCAGTTCCATGT-3'
Hspa5	Forward: 5'- AGCCCACCGTAACAATCAAG-3'
	Reverse: 5'- TCTTTTGTCAGGGGTCGTTC-3'
Actb (actin, beta)	Forward: 5'- CCACCATGTACCCAGGCATT-3'
	Reverse: 5'- GAAGGTGGACAGTGAGGC-3'
